# The Hemibiotrophic Apple Scab Fungus *Venturia inaequalis* Induces a Biotrophic Interface but Lacks a Necrotrophic Stage

**DOI:** 10.3390/jof10120831

**Published:** 2024-11-29

**Authors:** Ulrike Steiner, Erich-Christian Oerke

**Affiliations:** Institute of Crop Science and Resource Conservation—Plant Pathology, Rheinische Friedrich-Wilhelms-Universitaet Bonn, 53115 Bonn, Germany; u-steiner@uni-bonn.de

**Keywords:** hemibiotrophy, host response, pit fields, plasmodesmata, subcuticular development, transfer cells

## Abstract

Microscopic evidence demonstrated a strictly biotrophic lifestyle of the scab fungus *Venturia inaequalis* on growing apple leaves and characterised its hemibiotrophy as the combination of biotrophy and saprotrophy not described before. The pathogen–host interface was characterised by the formation of knob-like structures of the fungal stroma appressed to epidermal cells as early as 1 day after host penetration, very thin fan-shaped cells covering large parts of the host cell lumen, and enzymatic cuticle penetration from the subcuticular space limited to the protruding conidiophores. The *V. inaequalis* cell wall had numerous orifices, facilitating intimate contact with the host tissue. Pathogen-induced modifications of host cells included partial degradation of the cell wall, transition of epidermal cells into transfer cells, modification of epidermal pit fields to manipulate the flow of nutrients and other compounds, and formation of globular protuberances of mesophyll cells without contact with the pathogen. The non-haustorial biotrophy was characterised by enlarged areas of intimate contact with host cells, often mediated by a matrix between the pathogen and plant structures. The new microscopic evidence and information on the pathogens’ biochemistry and secretome from the literature gave rise to a model of the lifestyle of *V. inaequalis*, lacking a necrotrophic stage that covers and explains its holomorphic development.

## 1. Introduction

*Venturia inaequalis* (Cooke) G. Winter (1875) is an ascomycetous fungus, class Dothideomycetes, that causes apple scab, the most important disease in apple production worldwide [1]. The pathogen described as a hemibiotroph has remarkable features, as (I) it growths subcuticularly without penetrating host cell walls; (II) in its parasitic stage on young leaves and fruits, it neither kills plant cells nor produces haustoria within host cells for nutrient uptake; and (III) it perforates the cuticle for conidia production and uses the increased cuticular transpiration for its nutrition [2]. As germ tubes and subcuticular hyphae are non-melanised and conidia become melanised only in the later stages of their development, host plant cells have contact with non-melanised *V. inaequalis* cell walls only, despite the production of melanised conidia and ascospores [3]. The growth of scab lesions is limited in time (and space) due to ontogenetic resistance, which becomes complete by the time the leaves are fully expanded [4,5]. All apple cultivars exhibit ontogenetic resistance, which appears never to have been overcome by the pathogen [6,7]. In the late stages of the vegetation period of its host plant, *V. inaequalis* resumes hyphal growth, saprophytically colonises dead leaf material by melanised hyphae, and produces pseudothecia on leaf litter for its sexual stage, ascospore dissemination, and new host-plant infections in spring.

In addition to *V. inaequalis*, subcuticular pathogens include other species of the genus *Venturia*, e.g., *V. asperata* on apples [8], *V. effusa* on pecans [9], *V. nashicola* on Asian pears [10], *V. oleaginea* on olives [11], *V. paralias* on *Euphorbia* [12], *V. pyrina* on European pears [13], *Diplocarpon rosae* causing black spots on roses [14,15], and *Rhynchosporium secalis* causing scalding of barley and rye [16,17]. Nevertheless, there are differences between structures (and lifestyles) within the genus *Venturia*, e.g., *V. carpophila* on peaches produces melanised epicuticular hyphae [18], whereas the subcuticular runner hyphae of *V. inaequalis* are non-melanised.

Mechanical pressure is not required, but enzymatic hydrolysis is proposed to allow cuticle penetration [19]. Extracellular cutinase activity has been implicated, as this enzyme is produced by germinating conidia, and a cutinase inhibitor can prevent penetration [20,21]. The enzymatic activity is discussed as being localised and limited by the melanised ring structure at the interface between the appressorium and the plant surface [22]. Subcuticular-growing pathogens like *V. inaequalis* differentiate specialised subcuticular infection structures—primary stroma, runner hyphae, secondary stromata—in close contact with the underlying epidermal host cells [4,23]. These non-melanised hyphae differ from epicuticular germ tubes and hyphae as well as from hyphae produced in artificial cultures. Runner hyphae enable the subcuticular spread from the penetration site and are the initials for secondary stromata. They are reported to be wider and flatter than tubular hyphae and may form ‘hyphal superhighways’; stromata are mono- and multilayered pseudo-parenchymatic structures thought to be involved in nutrient uptake and effector delivery [24]; however, they also give rise to conidia. Secondary stromata produce melanised conidiophores that bulge out of the cuticle before forming conidia outside the plant tissue [3]. Damage to leaf cuticles is strictly limited to the pores necessary for the emerging conidiophores to release their conidia into the ambient air [2].

As the subcuticular hyphal growth of *V. inaequalis* is strictly apoplastic between the cuticle and epidermal cell layers, and without the formation of specific structures entering the plant cells (like haustoria), the sourcing and uptake of nutrients are hardly understood. Like obligate pathogens, *V. inaequalis* influences hormone levels at infection sites, specifically cytokinins, in its biotroph stage, most probably to enable biotrophic nutrition [25,26]. Cytokinin accumulation at infection sites affects the sink-source relations of the plant and results in the translocation of nutrients to the fungus [25].

Apart from cuticle penetration during infection, the host tissue exhibits no obvious damage throughout stroma development. Most damage of the apple tissue has been reported to result from breaching of the cuticle upon sporulation [5,27]. At this time, the epidermal cells underlying the stroma are reported to progressively become depleted of plastids and cytoplasm, accompanied by increasing vacuolation, leading ultimately to necrosis, which is possibly affected by partial cell wall degradation late in the infection cycle [23,28]. However, collapse of epidermal apple cells has rarely been detected in highly compatible *M. domestica*–*V. inaequalis* interactions [2,29]. In contrast, the epicuticular formation and release of masses of conidia requires the re-penetration of the plant cuticle by *V. inaequalis* conidiophores—two to three magnitudes the frequency of penetration sites covered by the tightly attached appressoria—which causes an increased cuticular transpiration, a crucial impact on the water status of the host plant [2].

For in vitro growing hyphae of *V. inaequalis*, the production and secretion of cellulase, β-glucosidase, pectinase, and endo- and exo-polygalacturonase activities have been described [5,30,31]. Provided these enzymes are only expressed late in infection with host cell wall degradation, they are unlikely to play a major role in nutrient acquisition during the establishment of infection [7]. In liquid cultures, *V. inaequalis* also produces melanoproteins [32,33]. A melanoprotein binding iron and copper has been demonstrated also in the host apoplast and has been discussed to be involved in the acquisition of ions and the release of cell-wall-degrading enzymes (CWDEs) [27,34]. They have been speculated to tether CWDEs and to facilitate their slow release [35], and to be involved in diverting the solute flow towards the site of infections, probably by altering the membrane permeability and solute transport system of apples to facilitate the availability of nutrients for pathogen growth and development [5]. However, as subcuticular hyphae of *V. inaequalis* are not melanised, a role of melanin in the acquisition of nutrients is very unlikely.

Plant–pathogenic fungi may modify their cell wall during host colonisation in order to avoid detection [36,37]. One strategy is to deacetylate chitin to chitosan [38,39,40]. Chitin and other β-glucans are MAMPs—strong elicitors of the plant immune system [41,42]. During subcuticular growth, *V. inaequalis* down-regulates genes for the biosynthesis of chitin and β-1,6-glucan and coats its infection structures with chitosan [43,44], a carbohydrate reported to be less effective in PTI [45,46] and a weak substrate of plant chitinases [47].

The *V. inaequalis* secretome includes carbohydrate active enzymes (CAZymes), proteases, peroxidases, lipases, small secreted proteins (SSPs), and many with an unknown role [48,49,50,51]. SSPs account for 40–60% of the secretomes throughout all fungal lifestyles and many of them have been described as effector proteins in diverse diseases. More than 600 SSPs have been reported from the *V. inaequalis* genome [51,52,53]. Many of these effector candidates are unique to *V. inaequalis*, and a few of them are known to belong to extended gene families. These effectors are expressed in waves at different time intervals during the infectious process [43,48]. When interpreting these secretome data, the non-obligate biotroph lifestyle of *V. inaequalis* has to be respected; the repertoire of enzymes and SSPs may include proteins specific to one of both lifestyles, whereas others may be active in both life stages.

Wang et al. [54] classified *V. inaequalis* as a biotroph member of Dothideomycetes within Ascomycota, with 13,741, 1754, and 486 proteins in the proteome, secretome, and effectome, respectively. The number of genes encoding CAZymes—glycoside hydrolases (GHs), glycosyl transferases (GTs), polysaccharide lyases (PLs), carbohydrate esterases (CEs), auxiliary activities (AAs), and carbohydrate-binding modules (CBMs)—and the number and activity of secondary metabolite biosynthetic gene clusters—nonribosomal peptides, polyketides, and terpenes—were rated below average, except for AAs. The CE, GH, and PL superfamilies are also known as CWDEs due to their role in the disintegration of the plant cell wall by bacterial and fungal pathogens [55].

The chemical composition of the cell walls of apple leaves is unknown; in apple fruit skin the substrate of *V. inaequalis* when causing fruit scab; pectin is the predominant polysaccharide at 65%, compared with 3% for cellulose [56]. *V. inaequalis* CAZymes are optimally adapted to this cell wall composition, with pectin-specific CAZymes predominating; the two most numerous classes of CAZymes in the *Venturia* secretome, after those with cutinase activity (CE5 domains), are GH28 and GH43, with pectin as a substrate [51]. One GH28 enzyme and two putative polysaccharide lyases, that also have pectin as the target substrate [57,58], are up-regulated during infection. This lytic activity may contribute to nutrition via degrading the surface polysaccharides of the epidermal cells beneath stromata.

Proteins similar to the *Aspergillus oryzae* CutL1 polyesterase/cutinase and HsbA are well represented in the *Venturia* infection secretomes [51]. Extracellular cutinase is produced by germinating *V. inaequalis* conidia, and a cutinase inhibitor can prevent penetration [21]. The high number of carbohydrate esterases in the *Venturia* secretomes is in agreement with an enzymatic penetration of the cuticle by *V. inaequalis* [51]. The hypothesis that cuticular degradation during host colonisation by *Venturia* fungi also makes the cuticle or cuticle precursors a source of nutrients for the pathogens [7] has to be revisited in view of the time and spatial limitation of cuticle degradation.

Cutin consists of omega hydroxy acids and their derivatives, which are interlinked via ester bonds, forming a polyester polymer of indeterminate size. Pectin is an acidic heteropolysaccharide in the primary and middle lamella of cell walls of terrestrial plants, with galacturonic acid as the main component. It may be degraded by pectinases; as a consequence, the middle lamellae break down and cells become separated from each other—a process not described for *V. inaequalis* infections so far. Pectinases catalyse either depolymerisation (hydrolases and lyases) or de-esterification (esterases) reactions. An endo-polygalacturonase of *V. inaequalis* has been characterised by Kollar [31]. The restriction of fungal growth to the subcuticular space suggests that pectinases released by infection hyphae or subcuticular hyphae may be important in infection [10]. Also, cellulase activity is induced in *V. inaequalis* in vitro and in vivo [59]. As cutin, pectin, and cellulose, the main components of the plant cell wall, do not contain nitrogen atoms—pathogens that degrade only the cell wall, ignoring embedded or apoplastic proteins— they lack a source of nitrogen essential for fungal growth and development.

Hardham [60] summarised the fungal strategies to gain access to the nutrients they need for growth, development, and reproduction: Necrotrophic fungi obtain nutrients by killing the host cells, forming expanding necrotic lesions. Biotrophic pathogens establish a close and stable relationship with living host cells and redirect the flow of nutrients from the plant cell into the pathogen. They form specialised feeding structures that take the form of haustoria or differentiated intracellular hyphae. Hemibiotrophs initially establish a biotrophic interaction with living host cells before killing the plant cells and turning to a necrotrophic lifestyle.

A key feature of scab pathogens is their ability to differentiate specialised subcuticular infection structures that, to date, remain largely understudied [44]. The subcuticular hyphae of *V. inaequalis* are not in contact with the plasma membranes of epidermal cells of apples but are likely to collect nutrients in the intercellular space. This lifestyle requires (I) *V. inaequalis* structures for nutrient interception; (II) modifications of apple leaf tissue for the release of nutrients for *V. inaequalis*, most probably induced by effectors of the pathogen. Therefore, light microscopy, scanning electron microscopy, and transmission electron microscopy were used to study the host–pathogen interface of apple leaf tissue and subcuticular hyphae of *V. inaequalis* in detail. The objectives were the identification of (I) fungal structures specialised for nutrient uptake that should not impair the flow of water, the medium transporting organic nutrients to infection sites, e.g., differentiation of hyphae to maximise the surface area in contact with the surface of plant cells/the nutrient broth of the apoplastic intercellular fluid; (II) modifications of plant cells—colonised epidermal cells as well as more remote mesophyll cells—able to promote a flow of nutrients to the immediate surroundings of subcuticular hyphae.

## 2. Materials and Methods

**Plant material**. Seedlings of apples (*Malus* × *domestica* Borkh., cvs. Golden Delicious, Cripps Pink) were grown in pots (9 cm × 9 cm × 8 cm) filled with standard potting mixture (Klasmann-Deilmann GmbH, Geeste, Germany) at 20 ± 2 °C and 16 h daylight (300 µmol m^−2^ s^−1^, Philips SGR 140, Hamburg, Germany) in a glasshouse with 50 to 70% relative humidity (RH). Plants were irrigated and fertilised (liquid fertiliser Flory 2 special, NPK 16 + 9 + 22) as required. Plants with at least four fully expanded leaves were used for the experiments. Inoculated plants were maintained at 20/18 °C (day/night), which is most favourable for rapid scab development, in a climate chamber (RH 50 to 70%).

**Pathogen and inoculation**. *Venturia inaequalis* (Cooke) G. Wint., isolate HS1, was cultivated on apple leaves by spraying conidia suspensions onto the leaves and incubating the plants at 100% RH for 48 h. Leaves with sporulating scab lesions were stored at −18 °C as inoculum. Conidia were washed off the stored leaves with tap water, passed through two layers of cheese cloth and adjusted to a conidia concentration of 1.0 × 105 conidia mL^−1^ using a Fuchs–Rosenthal hemacytometer. Apple leaves were spray-inoculated using a commercial hand sprayer (0.5 mL per leaf). Depending on the research question, the adaxial and abaxial side, respectively, were sprayed with inoculum before incubation at 100% RH for 48 h. Subsequently, RH was reduced to 50 to 70% until the end of the experiment. Four to eight plants per treatment were inoculated and used for sampling for microscopy.

**Microscopy**. All microscopic investigations were repeated several times (>3 times). For each experiment, samples for microscopic investigations were taken from similar leaves from at least 4 different apple plants. The images are representative for apple leaf tissue and the fungal structures indicated.

**Light microscopy**. Leaf discs were excised from the 2nd- to 3rd-youngest leaves of the apple plants by using a cork borer (Ø 1.1 cm). They were cleared in saturated chloral hydrate (Sigma-Aldrich, Darmstadt, Germany) or Farmer’s Fixative (95% ethanol/glacial acetic acid, 3:1) for 3 to 5 days. Trypan blue staining was carried out by boiling leaf fragments (1 cm^2^) in a mixture of phenol, lactic acid, glycerol, and distilled water (1:1:1:1) containing 1 mg mL^−1^ trypan blue for 1 min. Then, the tissue was cleared overnight in chloral hydrate. For brightfield microscopy, specimens were stained with acid fuchsin (Merck, Darmstadt, Germany; 0.01% in lactophenol) or aniline blue according to Bruzzesse and Hasan [61] for protein staining. Semi-thin sections were stained with toluidine blue (0.5 g toluidine blue, 0.5 g sodium tetraborate, 50 mL bidistilled water). For fluorescence microscopy of fungal structures and callose, leaf samples were rinsed with distilled water and stained either with Uvitex 2B (0.05%; Polysciences, Warrington, PA, USA) or with aniline blue (0.05% aniline blue in 0.067 M K_2_HPO_4_) according to Hood and Shew [62]. For imaging of total preparations and conidia, samples were studied under the light microscopes DMRB and DM6000 B (Leica, Wetzlar, Germany), equipped with Nomarski interference contrast and epifluorescence, respectively. The aniline-blue-stained pathogen structures were visualised using filter cube A (excitation 340–380 nm, beam splitter 400 nm, stop filter LP 425 nm). Images were recorded and analysed using the software Diskus (v. 4.60.1611; Technisches Buero Hilgers, Koenigswinter, Germany).

**Scanning electron microscopy (SEM)**. The preparation and examination of apple leaf samples were carried out according to the protocol of Juraschek et al. [63] for grapevine leaves. For studying subcuticular fungal structures, the leaf cuticle was removed by using adhesive tape. In some cases, the removed surface (on the tape) was investigated after sputtering.

**Transmission electron microscopy (TEM)**. The preparation and examination of samples were carried out as described by Schumacher et al. [64].

## 3. Results

### 3.1. Hypothesis 1: Formation of Pathogen Structures Suitable for Nutrient Uptake

#### 3.1.1. Primary Stroma and Runner Hyphae

**Figure 1:**
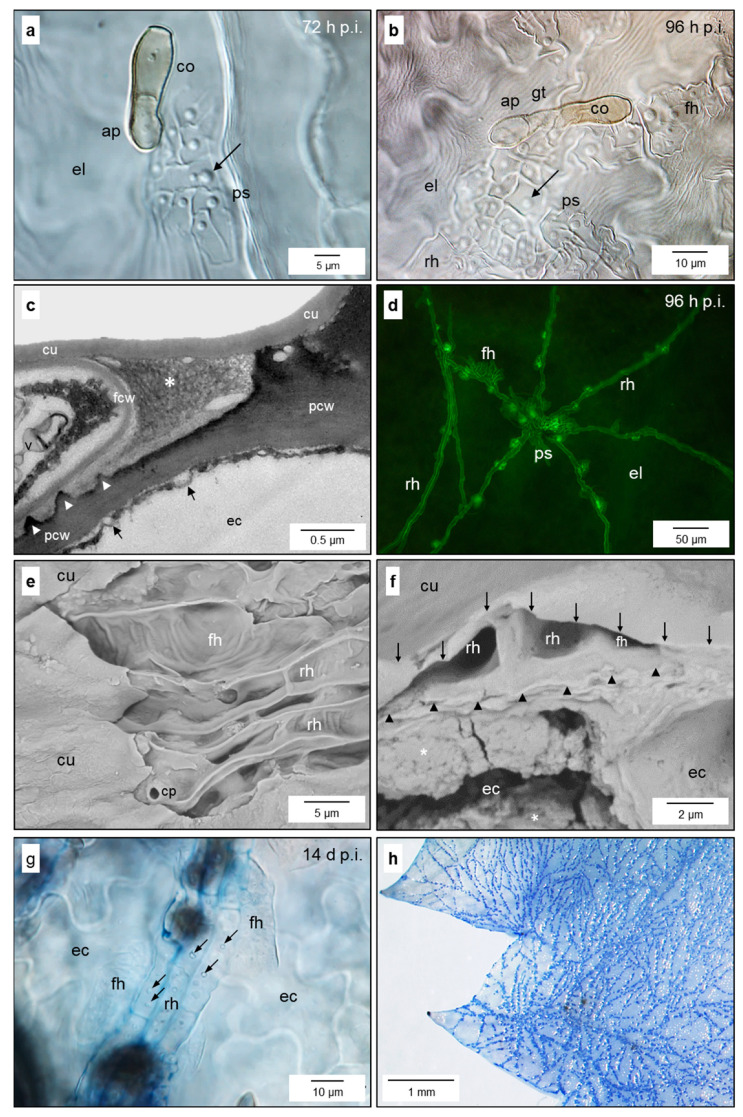
Formation of early subcuticular infection structures by *V. inaequalis*. Primary stroma (ps) 72 h p.i. (**a**) and 96 h p.i. (**b**) with knob-like structures (arrows) at the interface with the epidermal layer; details of the advancing hyphal tip between cuticle (cu) and cell wall (pcw), which is partially degraded, matrix material (asterisk) at the hyphal tip, cavities (arrow heads) at the interface, formation of vesicles at the plant cell membrane (arrows) (**c**); subcuticular spread from the primary stroma by runner hyphae (rh) that start to form lateral fan-shaped hyphae (fh) 96 h p.i. (**d**); runner hyphae, early fan-shaped hyphae, and initial of conidiophore (cp) after partial removal of the plant cuticle (**e**); cross-section of runner hyphae between plant cuticles (marked by ↓) and upper cell walls (arrow heads) of epidermal cells (ec) showing massive appositions of the cell wall (asterisks) (**f**); runner hyphae with knob-like structures (arrows) and lateral fan-shaped hyphae 14 d p.i. (**g**); spread of runner hyphae on minor leaf veins at the tip of a young apple leaf 7 d p.i. (**h**). Bright-field microscopy (**a**,**b**,**g**) [staining with trypan blue], transmission electron microscopy (**c**), fluorescence microscopy after aniline blue staining (**d**), scanning electron microscopy (**e**,**f**), RGB camera, ink staining (**h**). ap, appressorium; co, conidium; el, epidermal layer; gt, germ tube.

Cuticular penetration from epicuticular appressoria gave rise to a primary subcuticular stroma 2 to 3 days post inoculation (d p.i.) (Figure 1a,b). As early as 72 h p.i., the cells of this monolayer showed knob-like structures at the interface with the epidermal host cells beneath—indicated by the common optical level and central orifice of some of these structures (Figure S1a). At the hyphal apex, the cuticle was separated from the cell wall, which was partially degraded; a maximum of half of the cell wall thickness was affected (Figure 1c) and in many cases no degradation was observed. The interface between the pathogen cell wall and host epidermis exhibited a (rippled) matrix of varying thickness.

For the subcuticular spread of the pathogen, primary stroma produced runner hyphae with a diameter wider than that of the epicuticular germ tubes (7–9 µm vs. 5–6 µm) and also showing knob-like structures at the interface with epidermal cells (Figure 1d,g and Figure S1c). Often, several hyphal strands grew side-by-side (Figure 1e,f). Runner hyphae produced a hyphal network starting on the minor veins and large plated areas of the leaves before the emergence of first conidia (Figure 1h and Figure S1d).

#### 3.1.2. Fan-Shaped Hyphae

**Figure 2:**
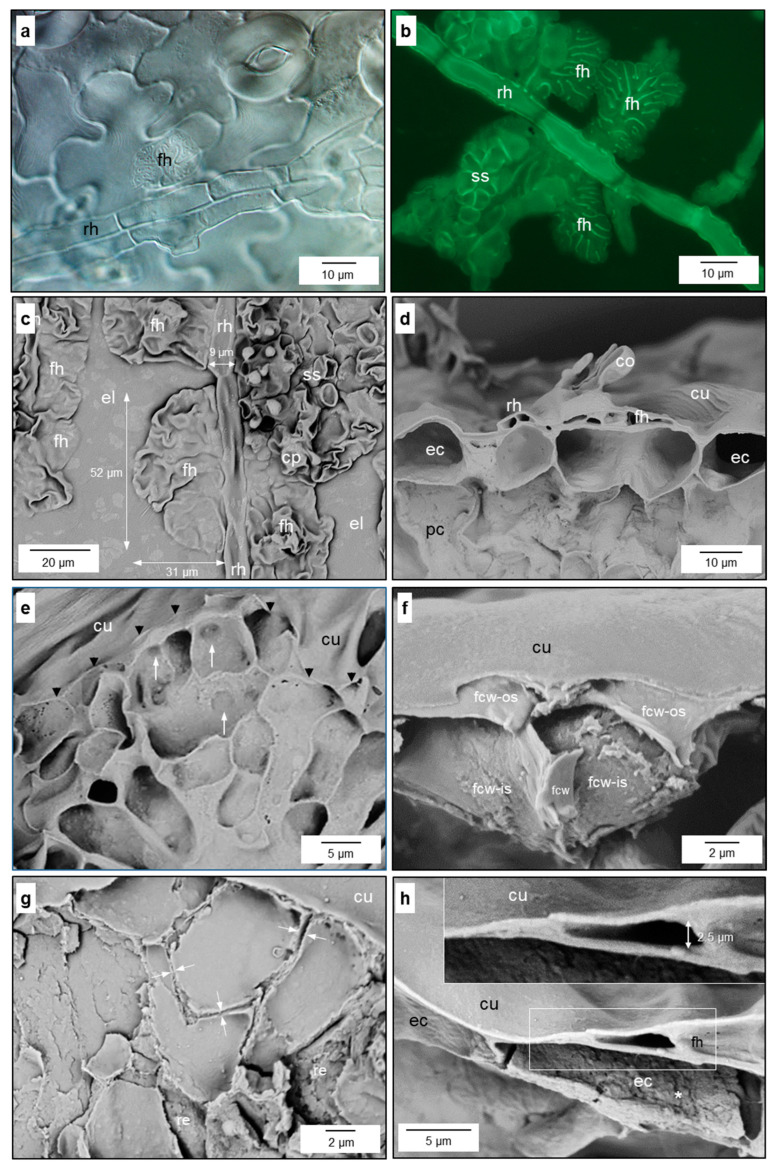

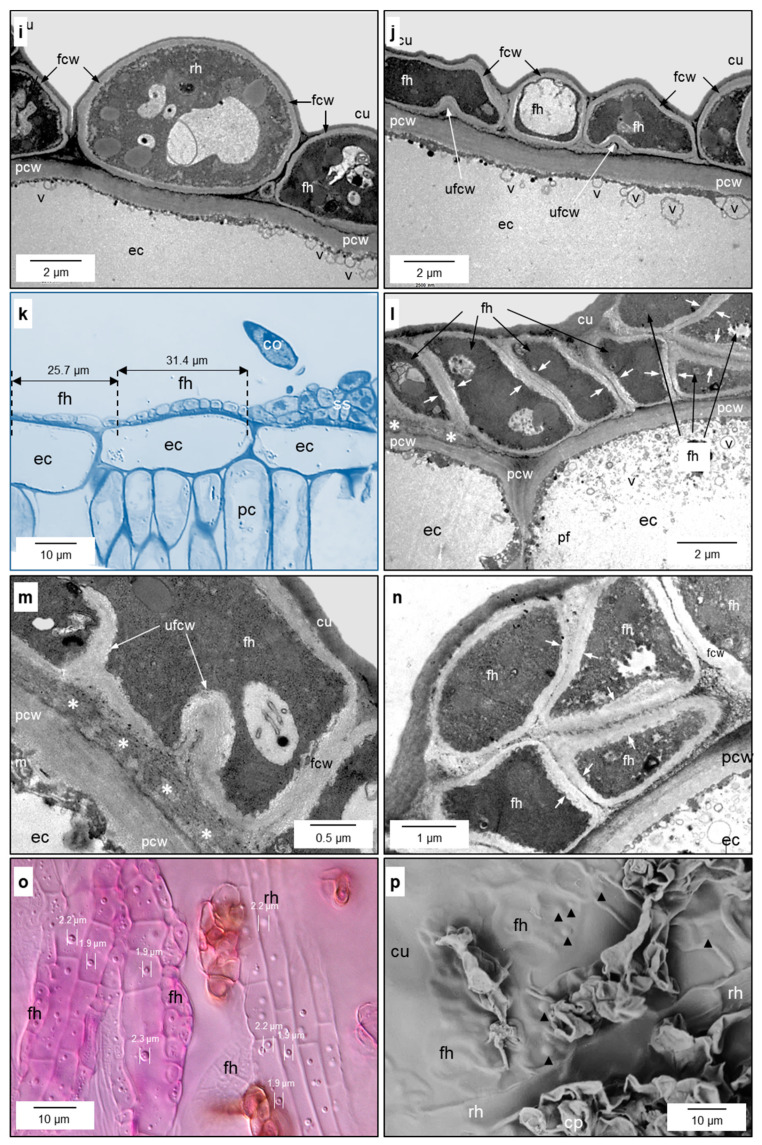
Subcuticular formation of fan-shaped hyphae (fh) by runner hyphae of *V. inaequalis*. Early fan-shaped hyphae laterally expand from a cell of a runner hypha (rh) on the lumen of an epidermal cell (**a**); various sizes and stages of fan-shaped hyphae expanding from a runner hypha; fungal cell walls stained with aniline blue (**b**); dimensions of fan-shaped hyphae without and with conidiophores (cp) formed in later developmental stages (top view; (**c**)); lateral view of infection site with runner hyphae, fan-shaped hyphae, and conidiophores with conidia (co) on top of epidermal cells (ec) and covered by the cuticle (cu) (**d**); top view of fan-shaped hyphae after (partial) removal of plant cuticle (arrow heads mark cuticle margin); arrows indicate vents in the fungal cell wall (**e**); lateral view of hyphal cells advancing underneath the cuticle; the outer surface (fcw-os) and the inner surface (fcw-is) of the fungal cell wall (fcw) demonstrate the low-rise structure (**f**); delineation of fungal cells of fan-shaped hyphae by two cell walls at the contact zone (after partial cuticle removal) (**g**); tip of fan-shaped hypha advancing above an epidermal cell to build the subcuticular space (**h**); section of runner hypha with neighbouring fan-shaped hyphae (**i**); formation of mem-brane vesicles by the epidermal cell underneath fan-shaped hyphae of. *V. inaequalis* (**j**); expansion of fan-shaped hyphae limited to the lumen of individual epidermal cells (**k**); ultrastructure of fan-shaped hyphae above epidermal cells with a pit field (pf) and increased formation of vesicles (**l**); undulations of the fungal cell wall (ufcw) and matrix material (*) between cell walls of pathogen and plant, respectively (**m**); the cell wall of the contact area of fungal cells was thicker, but less compact than the cell wall in contact with epidermal cell and cuticle, respectively (**n**); knob-like structures at the interface between the subcuticular pathogen and epidermal cell after staining with fuchsin acid (**o**) and in SEM image (**p**). Bright-field (**a**,**k**) [staining with toluidine blue], o [staining with fuchsin acid], and fluorescence (**b**) microscopy, scanning electron microscopy (**c**–**h**,**p**), transmission electron microscopy (**i**,**j**,**l**–**n**). pc, palisade cell; ss, secondary stroma.

As early as 3 to 4 d p.i., the long runner hyphae laterally differentiated into ramified, fan-shaped, ultra-thin hyphae covering large areas of the epidermal cell surfaces beneath (Figure 2a–c). The small and flat cells (height 1–2 µm) were branched several-fold and tapered to the margin that advanced beneath the cuticle (Figure 2f,h,k). Undulation of the fungal cell wall on the epidermal cell increased the surface of the interface (Figure 2j,m) and anticlinal cell walls of fan-shaped hyphae often were thicker than periclinal walls (Figure 2l,n). The fan-shaped structures were produced above the lumen of epidermal cells and rarely exceeded the next anticlinal wall of the epidermal layer (Figure 2e,k). Their lobes/thin constituents were separated from each other as demonstrated by the existence of two cell walls of adjacent hyphae (Figure 2g). The small cells formed a structure with a large surface-to-volume ratio, optimal to capture and take up nutrients from the intercellular fluid like a filter/strainer (Figure 2e,k,m). These fan-shaped hyphal structures on epidermal cells did not act as a barrier but are likely to be porous/permeable for water and substances dissolved within. Similar to primary stromata and runner hyphae, early fan-shaped hyphae had numerous knob-like structures at the interface with epidermal cells (Figure 2o,p).

**Figure 3:**
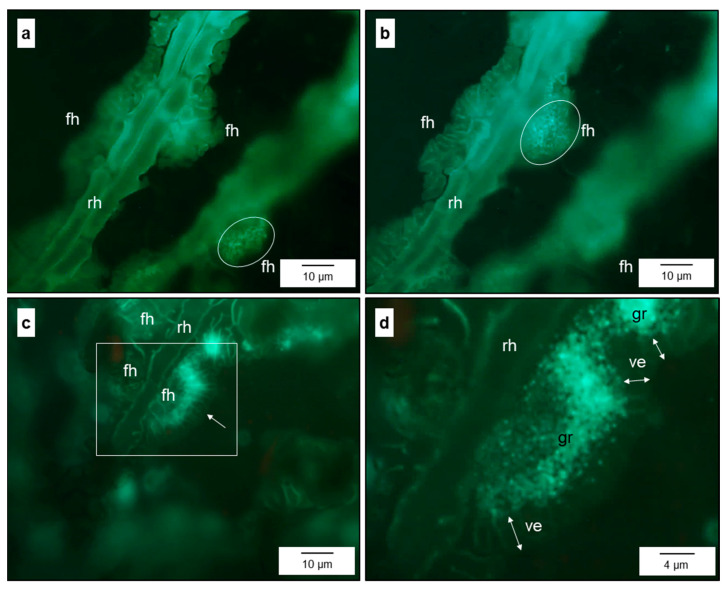
Formation of fan-shaped hyphae at runner hyphae of *V. inaequalis*. Early stage of fan-shaped hyphae (fh) with formation of fluorescent granules at the plant–pathogen interface (**a**,**b**); focus level of b below that of (**a**); later stage with fluorescent granules (gr) at the plant–pathogen interface surrounded by a verge (ve) free from granules (**c**,**d**) [detail of (**c**)]). rh, runner hypha.

**Figure 4:**
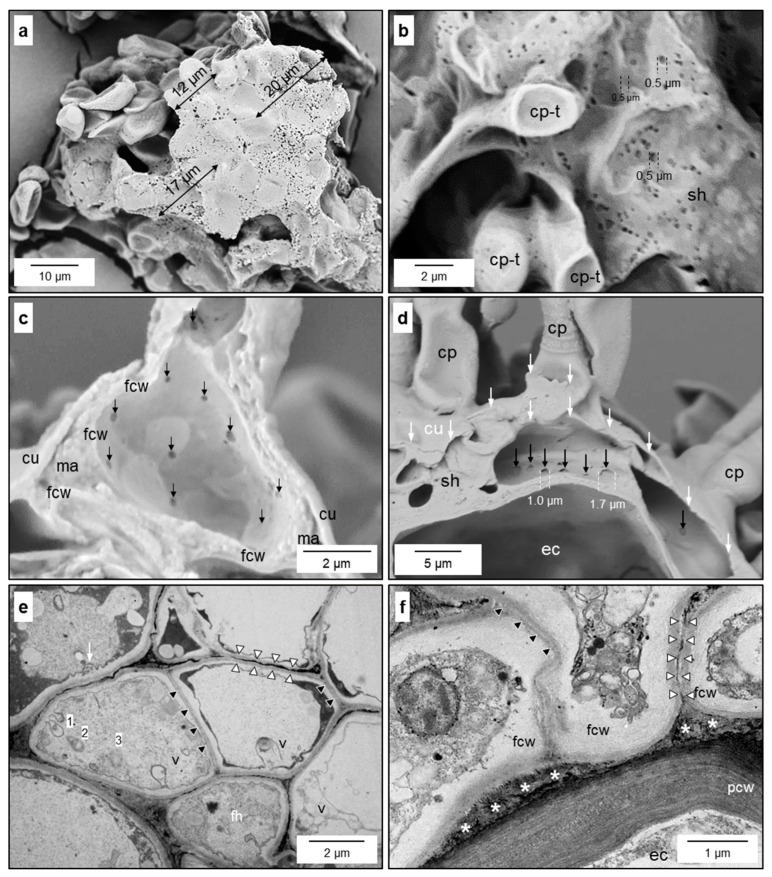
Morphological details of the subcuticular *V. inaequalis* hyphae. Heterogeneous pattern of cell wall orifices at the lower surface of the subcuticular stroma in contact with host epidermal cells [after dislodging of the epidermal layer] (**a**); top view on the surface of fungal secondary stroma with cell wall orifices [after dislodging of the cuticle] (**b**); section through a conidiophore with several small orifices (↓) at the inner surface of the fungal cell wall (fcw; (**c**)); subcuticular hypha (sh) above an epidermal cell (ec) with larger orifices at the interface with the epidermal cell (**d**); vesicle trafficking in the apex of a subcuticular hypha surrounded by other hyphae; 1, 2, 3, stages of vesicles (v) during endocytosis; hyphal septae (black arrowheads) and two cell walls of neighbouring hyphae (white arrowheads) (**e**); deposition of debris material (*) at the interface between the plant cell wall (pcw) and fungal cell wall (fcw; (**f**)). cp-t, top of conidiophore; cu, cuticle; ma, matrix between cuticle and conidiophore; cp, conidiophore. Scanning electron microscopy (**a**–**d**), transmission electron microscopy (**e**,**f**).

Focussing even more closely on the pathogen–host interface, the contact zone between fan-shaped hyphae (FH) and the epidermal cell wall was characterised by the presence of granules fluorescing after staining with aniline blue, which were surrounded by a verge (sealing lip) of FH—thin outer lobes of the FH structure on the epidermal cells (Figure S1e,f and Figure 3). The number of fluorescing granules increased with the size (and age) of the FH (Figure 3b,d). These granules were limited to the FH and could not be detected beneath runner hyphae. Electron microscopy revealed that FH at later stages of pathogenesis were surrounded not only by the nutrient broth of the apoplast but also by residual material (Figure 2m and Figure 4f). The spatial pattern of runner hyphae and fan-shaped hyphae upon the cells of the epidermal layer was spatially oriented in order to maximise the contact area with the pit fields of epidermal cells beneath.

#### 3.1.3. Secondary Stromata

Two to three days after their formation, fan-shaped hyphae often developed into multilayered secondary stromata. In the case of the colonisation of intercostal epidermal cells, secondary stromata had three to four layers, whereas infections above leaf veins and of stem tissue could result in the formation of more layers (Figure 4e and Figure S1). Conidiophores were the only melanised fungal cells and were able to penetrate the cuticle from the subcuticular space to the outside. The secondary stroma of *V. inaequalis* was formed by aggregations of hyphae with septae with simple pores (Figure S1h). Experimental removal of the plant cuticle revealed that the fungal cell wall with contact to the epidermal cell wall (lower interface), as well as with the cuticle (upper surface), exhibited numerous notches/orifices visible in SEM images (Figure 4a,b,d). Images of the inner cell wall of *V. inaequalis* confirmed the existence of smaller orifices of varying diameter (Figure 4c,d). Within the subcuticular hyphae, vesicle trafficking crucial for the import of nutrients (endocytose) and the export of effectors (exocytose) could be observed (Figure 4e). TEM images exhibited the unipolar growth of fungal hyphae with a common septum within hyphae and two separate cell walls between adjacent hyphae (Figure 4f and Figure S1h).

**Figure 5:**
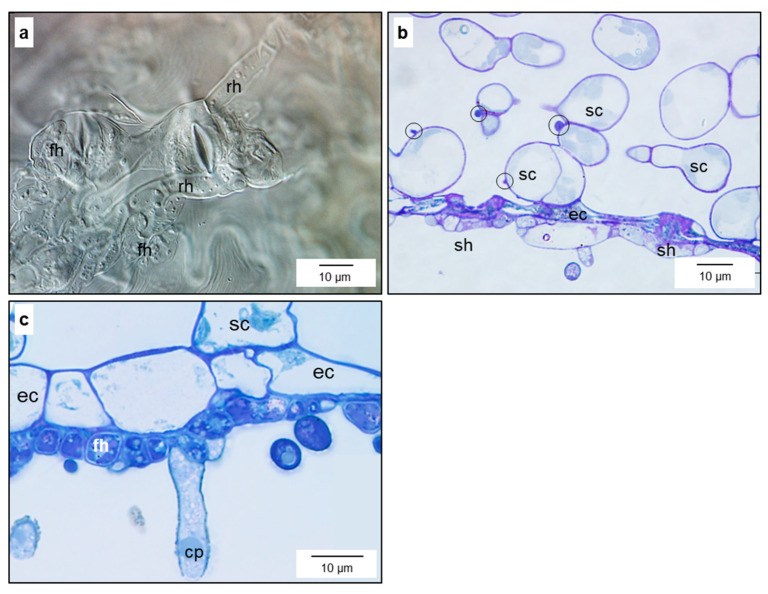
Colonisation of the abaxial epidermal layer by *V. inaequalis*. Formation of runner hyphae (rh) and fan-shaped hyphae (fh) in immediate vicinity of a stoma (**a**); development of subcuticular hyphae (sh) affecting the epidermal cell layer; cells of the spongy parenchyma (sc) respond to infection by the formation of globular protuberances stained blue (circles; (**b**)); subcuticular hyphae with formation of a conidiophore (cp) hardly affecting the epidermal layer (**c**). Scanning electron microscopy (**a**); bright-field microscopy of semi-thin sections stained with toluidine blue (**b**,**c**). ec, epidermal cell.

During all stages of leaf colonisation, the plant cuticle covered all pathogen structures except mature conidiophores. This was true for infections of the adaxial as well as for the abaxial leaf side (Figure 5a–c). The integrity and functionality of the plant cuticle was preserved and even fixed by the pathogen in case the abaxial epidermal layer was at risk of collapsing and losing its function as a water barrier during fungal colonisation (Figure 5b).

### 3.2. Hypothesis 2: Modifications of Apple Leaf Tissue in Response to Venturia inaequalis Colonisation

#### 3.2.1. Plasmodesmal Network of Epidermal Cells

**Figure 6:**
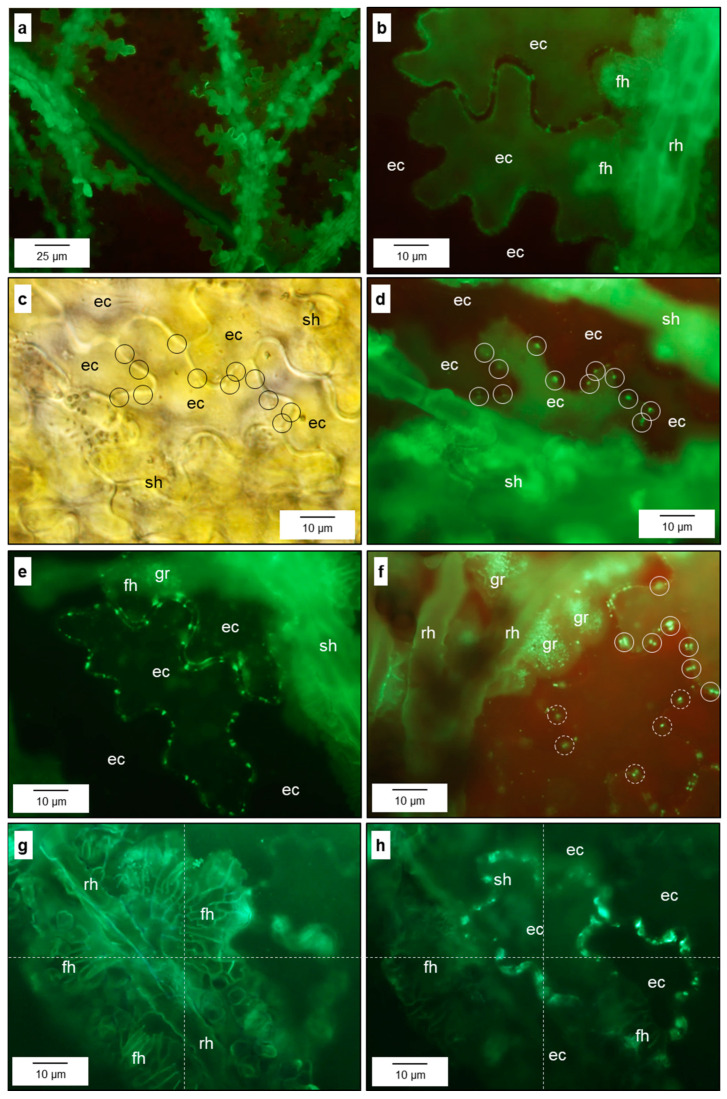
Effect of *V. inaequalis* colonisation on anticlinal pit fields of epidermal cells of apple leaves. Fluorescence of epidermal cells underneath subcuticular hyphae after staining with aniline blue (**a**); formation of callose at pit fields of neighbouring epidermal cells (ec) in immediate vicinity of runner hyphae (rh) and fan-shaped hyphae (fh) above (**b**); spatial pattern of pit fields of an epidermal cell (**c**) and callose deposition at pit fields (**d**); callose accumulation at pit fields of an epidermal cell next to a cell colonised superficially (**e**); variation in the intensity of callose accumulation at pit fields varying from strong (solid line) to light (dotted line) (**f**); spatial pattern of subcuticular colonisation (**g**) and callose accumulation at the pit fields of epidermal cells beneath (**h**). Bright-field microscopy (**c**); fluorescence microscopy (**a**,**b**,**d**–**h**) after staining with aniline blue. gr, fluorescent granules; sh, subcuticular hyphae.

Pit fields of anticlinal epidermal cells harbouring primary plasmodesmata were visualised by callose staining of plant cell walls with aniline blue and focussing on the respective level of middle lamellae (Figure 6). Epidermal cells had about 20 (n = 11) pit fields with plasmodesmata in one optical level connecting them to their horizontal neighbours (Figure 6c,d and Figure S2a,b). The average distance between pit fields was 7 µm. The intensity of callose accumulation in pit fields of epidermal cell walls varied and reduced the capacity of plasmodesmata in horizontal transfer (Figure 6b,e,f).

Callose accumulation at pit fields occurred not only beneath subcuticular pathogen structures, but also in adjacent epidermal cells, indicating a plant reaction which was not successful in limiting the pathogenesis of *V. inaequalis* (Figure 6b,g,h). As demonstrated by a varying degree of fluorescence staining, the permeability of the cytoplasm membrane of affected epidermal cells was modified (Figure 6a,b).

#### 3.2.2. Plant–Pathogen Interface

**Figure 7:**
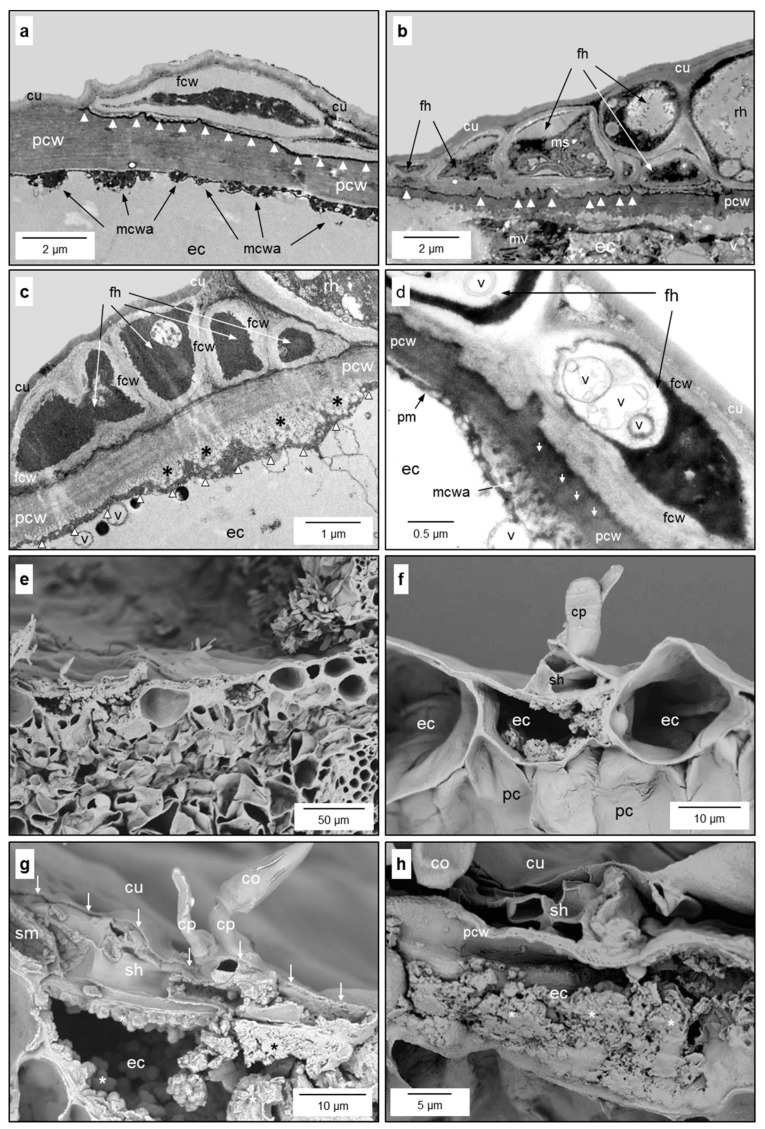
Reaction of epidermal cells of apple leaves to subcuticular development of *V. inaequalis*. Increased formation of cytoplasm membrane vesicles in an epidermal cell (ec) underneath a subcuticular hypha (**a**); formation of membranous vesicles (mv) in an epidermal cell and membrane staples (ms) in the fan-shaped hyphae of *V. inaequalis* above, respectively; rippled host–pathogen interface (**b**); membranous apposition (*) and increased vesicle (v) formation at the plant cell wall underneath fan-shaped hyphae (fh); anticlinal fungal cell walls (fcw) thicker than periclinal walls (**c**); membranous cell wall appositions (mcwa) and loosening of the plant cell wall in contact with a subcuticular hypha (**d**); spatial pattern of epidermal cells with and without cell wall ingrowth in response to scab development (**e**); close-up of epidermal cells with and without cell wall ingrowth underneath infection structures of *V. inaequalis* (**f**); increase in the deposition of (membranous) material (*) in epidermal cells, superficially colonised by *V. inaequalis* in an early (**g**) and later (**h**) stage of pathogenesis. Images from transmission electron microscopy (**a**–**d**) and scanning electron microscopy (**e**–**h**), respectively. co, conidium; cp, conidiophore; cu, cuticle; pc, cell of palisade parenchyma; pcw, plant cell wall; pm, plasma membrane; rh, runner hypha; sh, subcuticular hypha; sm, subcuticular matrix material.

The outer cell wall of colonised epidermal cells revealed disaggregation across the wall, which is probably an indicator of a facilitated transport through the cell wall (Figure 7b,d and Figure S2f). The decreased compactness of the otherwise solid wall was limited to the plant–fungus interface (Figure S2g).

Intricate protuberances/ingrowth of the epidermal cell wall of some epidermal cells beneath subcuticular hyphae of *V. inaequalis* demonstrated a transformation into transfer cells (Figure 7). Modified cells were typically surrounded by epidermal cells without any modification. TEM images revealed membranous cell wall appositions of different intensities at the inner side of the outer epidermal cell wall SEM images (Figure 7a–d). In rare cases, epidermal cells responded to the presence of subcuticular hyphae with the formation of a solid cell wall apposition on the inner side (Figure S2h,i). Adjacent epidermal cells, however, exhibited intensive vesicle trafficking towards the growing pathogen above (Figure S2i). Also, SEM images revealed that only some epidermal cells beneath the subcuticular hyphae responded with the formation of massive modifications of the cell wall (Figure 7e–h). Both periclinal cell walls—the top in contact with the pathogen, the bottom forming the interface with mesophyll cells—were affected by cell wall depositions, whose intensity increased during pathogenesis.

**Figure 8:**
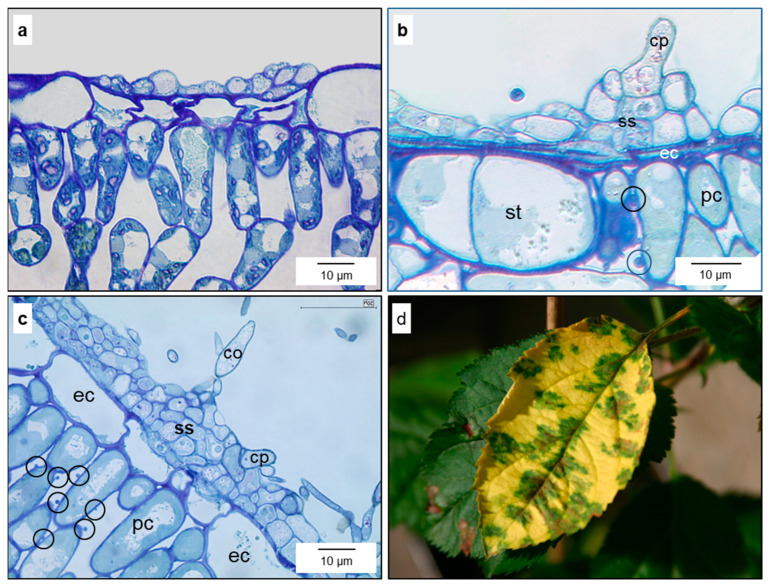
Effects of *V. inaequalis* on the morphology of apple leaf tissue in later stages of pathogenesis. Compression of colonised epidermal cells—fungal stroma with one and two layers, respectively; integrity of the cytoplasm of epidermal cells (**a**); formation of globular protuberances (circles) at the surface of a palisade cell (pc); epidermal cells (ec) beneath the sporulating stroma (ss) compressed, but not necrotic (**b**); multi-layered stroma with sporulation hardly affecting the morphology of epidermal cells of an apple stem 35 d p.i. (**c**); formation of green islands under scab lesions, which present as brown due to the massive presence of melanised conidia, not because of the formation of plant necroses (**d**). Bright-field microscopy of leaf sections stained with toluidine blue (**a**–**c**); RGB camera (**d**). co, conidium; cp, conidiophore.

Intensive subcuticular colonisation of leaves by *V. inaequalis* hyphae sometimes resulted in a turgor loss of epidermal cells, and in some cases the epidermal cell layer beneath secondary stromata of *V. inaequalis* was compressed (Figure 8a,b). A loss of cytoplasm integrity was never observed and often epidermal cells beneath hyphae maintained full turgescence (Figure 8c). The vitality of colonised leaf tissue was demonstrated by the formation of green islands under the sites of *V. inaequalis* sporulation, producing the brown, melanised conidia on the surface of apple leaves (Figure 8d). Islands of green leaf tissue beneath and around scab infections were observed on leaves infected for several weeks in apple orchards as well as under controlled conditions (5 weeks after inoculation).

#### 3.2.3. Response of Remote Cell Layers of Infected Apple Leaves

**Figure 9:**
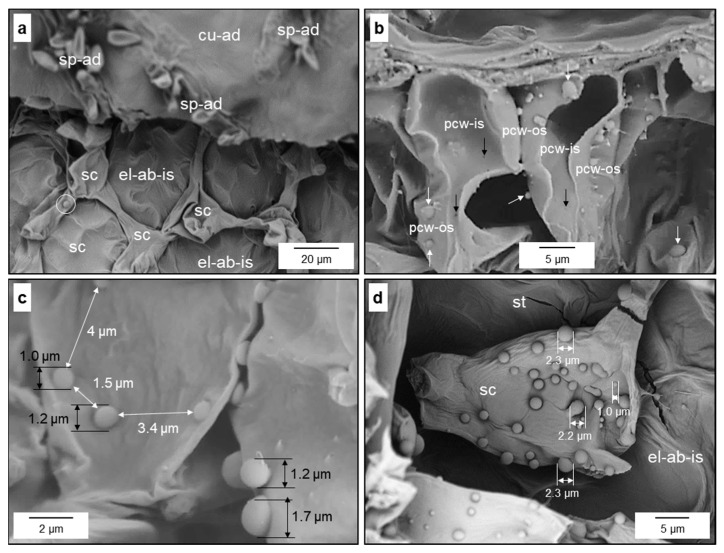
Modifications of mesophyll cells during *V. inaequalis* pathogenesis. Globular protuberance (circle) on the surface of a spongy parenchyma cell (sc); infection of adaxial epidermal cells with sporulation causes modifications of remote cells 16 d p.i. (**a**); mesophyll cells respond to subcuticular hyphae by the formation of globular, knob-like protuberances (white arrows) on the outer surface of cells of the palisade (pcw-os) and spongy parenchyma (white arrow bottom right); cross-section also visualising wall thinning spots (black arrows) on the inner surface of palisade cells (pcw-is), probably corresponding to protuberances at the outer side (**b**); size and number of knob-like structures on mesophyll cells varied; globular protuberances of palisade cells between adjoining cells and oriented to the intercellular space (**c**); individual spongy parenchyma cell intensively covered by protuberances with a diameter up to >2 µm (**d**). Images from scanning electron microscopy. cu-ad, adaxial cuticle; el-ab-is, inner surface of abaxial epidermal layer; sp-ad, adaxial sporulation; st, stoma.

Seven to ten days after infection, some mesophyll cells’ neighbouring epidermal cells subcuticularly colonised by *V. inaequalis* exhibited globular protuberances with a diameter of up to 2 µm on the cell surface (Figure 9). The formation started in the parenchyma layer adjacent to the infection site and often expanded to the spongy parenchyma and palisade parenchyma, respectively, during further infection. The intensity and size of these protuberances varied considerably. The close contact between palisade cells became loose and the protuberances were formed at both the interface of neighbouring cells as well as at the interface to the intercellular space (Figure 9c). The interior wall of affected palisade cells had small indentions, probably corresponding to the site of outer protuberances (Figure 9b). Some cells of the spongy parenchyma formed a high number of protuberances, with neighbouring cells showing no visible modification (Figure 9a,d).

## 4. Discussion

Subcuticular development of *V. inaequalis* is characterised by strictly apoplastic growth between the cuticle and epidermal layers without producing structures that enter plant cells and without causing obvious damage to apple leaf tissue. Runner hyphae and stromata are thought to be involved in nutrient acquisition and effector delivery [44].

Biotrophic Pathogen Structures

After cuticle penetration from epicuticular appressoria, the subcuticular colonisation of apple leaves by mono-layered primary stroma, thick runner hyphae, and laterally formed, thin, fan-shaped hyphae (FH) is intensive. Runner hyphae preferentially spread above anticlinal host cell walls—probably with a high density of pit fields—and produce lateral fan-shaped hyphae which, in early stages, form a monolayer positioned on top of the lumen of epidermal cells. Rarely, FH exceed the margin of the epidermal cell beneath and expand to adjacent cells. As especially the epidermal layer above leaf veins provides a high nutrient content of the intercellular fluid, the growth and sporulation of *V. inaequalis* is often stronger in veins than on intercostal leaf areas.

The small and very thin FH advance under the cuticle by separating it from the epidermal cell wall beneath, most probably by secretion of hydrolytic enzymes like polygalacturonases and other pectinases. They have a high surface-to-volume ratio, often even increased by undulations of the cell wall in contact with the plant cell, and cover large areas of the surface of epidermal cells. In later stages, FH become the foundation of multi-layered secondary stromata that produce melanised conidiophores and conidia [3]. FH form a layer of cells porous for water (and nutrients) which is considerably larger than the leaf area, with sporulating conidiophores perforating the plant cuticle from inside, and which is likely to function as a filter for nutrients, not as a water barrier. After cuticle perforation by *V. inaequalis* conidiophores, the secondary stroma contributes to the redirection of intercellular water and nutrients to the pathogen and the uptake of organic nutrients facilitated by indentions and orifices of the fungal cell wall. In the case of abaxial scab infections, the dense layer of fan-shaped hyphae may even support the functionality of the fragile abaxial epidermal cells as a layer regulating the loss of water to the ambient air.

The activity of fungal cutinases often described as crucial for pathogenesis [7,21] has to be limited in time and space to the penetration of the cuticle for invasion and for sporulation—essential for both pathogen spread and the redirection of water flow towards the subcuticular pathogen. *V. inaequalis* preserves the functionality of the plant cuticle by minimising its damage to the sites of emerging conidiophores [2]. Other hydrolases (pectinases, etc.) reported for *V. inaequalis* [30,31,51] are essential for the separation of cuticle and epidermal cell walls in order to produce the subcuticular niche.

The Host–Pathogen Interface

The huge interface between subcuticular fan-shaped hyphae and the adaxial side of epidermal cells facilitates the nutrient uptake of the pathogen. High metabolic activity is favoured by small cells. Not only is the abaxial side of the mycelium in contact with the nutrient mixture of the intercellular fluid provided by the plant cells, but all sides of the abutting fungal cells are able to contribute to nutrient uptake. Their straining activity is likely to be highest in anticlinal fungal cell walls, which are thicker than periclinal walls, although disaggregated and less compact.

The first stages of pathogenesis after penetration of the leaf cuticle—primary stroma, runner hyphae, and early fan-shaped hyphae—are likely to have rather low nutrient requirements, which may be matched by the nutrients available in the apoplast; however, knob-like structures of these hyphae appressed to host cells occur already in these early stages. These structures can be detected by using light and scanning electron microscopy and may be interpreted as areas of intimate contact between pathogen and host, probably related to notches in the fungal cell wall, facilitating the exchange of nutrients and effectors. As the subcuticular fungal cells are tightly surrounded by epidermal cell walls and the intact cuticle, respectively, small cavities (vents) in the cell wall do not affect the integrity of the pathogen. In later stages, especially during conidiation with high nutrient requirements, expanded fan-shaped hyphae produce a large interface with epidermal cells, which themselves have to be nourished by mesophyll cells—likely via an increased formation or modifications of secondary plasmodesmata. The realisation of modifications in the metabolism and morphology of plant cells induced by pathogen effectors is a series of events which takes some time.

Partial degradation is only one of the modifications of the cell walls of apple leaves. The areas in direct contact with *V. inaequalis* hyphae often appear loosened and weakened with anticlinal abnormities, and vesicle formation of the cytoplasm membrane is often increased. Some epidermal cells respond by the formation of membranous wall ingrowth, characterising transfer cells [65]. Their enhanced capacity for nutrient transport is conferred by an amplified plasma membrane surface area, enriched in membrane nutrient transporters, supported on an intricate wall labyrinth [66]. They are located at bottlenecks for nutrient transport between apoplastic and symplastic compartments, e.g., at sites of nutrient loading/unloading of vascular pipelines or interfaces between host and biotroph pathogens [66,67]. The formation of transfer cells is induced by external stimuli, e.g., light, or infection by nematodes [68,69] and rust fungi [70]. Adaxial epidermal cells of *Vicia faba* cotyledons undergo trans-differentiation to transfer cell morphology and function within hours [67]. In *Arabidopsis thaliana*, wall ingrowth formation in phloem parenchyma transfer cells occurring in leaf minor veins is induced by their phloem-loading activity, which is regulated by sucrose [71]. In apple leaves, leaf minor veins are the preferred sites of scab development. The fluffy membrane appositions at the inner cell wall of epidermal cells are different from solid cell wall appositions, which rarely occur as a non-successful resistance response of the host.

Fungi may develop structures similar to transfer cells, especially in the case of the Hartig net in ectomycorrhizae, where the penetrating hyphal system is a broad-lobed, fan-like hyphal front with rare cellular septation. The multinucleate coenocytic organisation has wall ingrowths which are actually incomplete septae [72,73,74]. The structure of the fan-shaped hyphae of *V. inaequalis* produced during subcuticular growth in apple leaves is very similar to the top view of the Hartig net of ectomycorrhizal fungi. Host root rhizodermal and cortical cells in contact with hyphae may also develop wall protuberances [75]. Both structures of the pathogen and changes in plant cells obviously increase the surface of the interface in a corresponding manner.

Pores Regulate the Flow of Nutrients and Information

The heterogeneity in the response of epidermal cells to subcuticular *V. inaequalis* hyphae is in agreement with observations that the intercellular movement of resources and information can be controlled by the density and structure of plasmodesmata, as well as by their opening status. Cell-to-cell connectivity within the epidermal layer is heterogeneous due to variation in the status—open, closed, intermediate—of plasmodesmata and leads to a mosaic of connectivity [76,77,78]. As plasmodesmata allow the distribution of resources and the passage of signals by hormones, small RNAs, transcription factors, and mRNAs, the restriction of these processes by callose accumulation reduces the interactions between plant cells and reorganises the flux of resources and information on a local scale. The induction of secondary plasmodesmata in the cell walls of epidermal cells in contact with the cuticle and the pathogen, respectively, would optimise the nutrient availability for *V. inaequalis*.

Plasmodesmata connect neighbouring cells to form the plant symplast (primary and secondary plasmodesmata); they are cytoplasmatic strands including the endoplasmatic reticulum. The respective cell wall is thin and forms pit fields with several plasmodesmata. Information on the number of plasmodesmata connecting epidermal cells to the palisade cells of apple leaves is lacking. In other plant species, the number differs between species but is similar to the number of anticlinal connections to other epidermal cells [79]. The number of plasmodesmata connections between palisade and spongy mesophyll cells is three to one-hundred times higher. It is not known whether epidermal cell walls in contact with the cuticle have plasmodesmata primordia (half-plasmodesmata) or pore-like structures facilitating the transport of vesicles or solutes through the cell wall to the exterior/apoplast, or whether these structures may be induced by *V. inaequalis*.

The number and density of plasmodesmata may be actively decreased or increased [80]. The formation of secondary plasmodesmata may be induced by phytohormones [81,82], flower-inducing day length [83], and parasitic plants which use this mechanism to develop new plasmodesmata between themselves and their hosts [84]. *Cuscuta* haustorial cells share plasmodesmata with hosts across chimeric cell walls [85,86], and these have been implicated in the host–parasite mobility of RNA [87]. In Arabidopsis, an increase in cell size increases and decreases the number of complex plasmodesmata of anticlinal and periclinal walls, respectively [81].

Being symplastic pores that facilitate cell-to-cell communication between neighbouring cells, plasmodesmata are a target of pathogen effectors [88]. The *Melampsora larici-populina* effector Mlp37347 accumulate exclusively at the plasmodesmata of *A. thaliana* and increase plasmodesmal flux by reducing callose deposition [89]. Effector Six5 of the wilt pathogen *Fusarium oxysporum* directly targets plasmodesmata [90,91] and the *Phytophthora brassicae* effector RxLR3 increases plasmodesmal flux by blocking the activity of the callose synthases CalS1, CalS2, and CalS3 at plasmodesmata [92]. Hofmann et al. [93] reported that a temporal callose deposition along plasmodesmata impaired the symplastic exchange in young syncytia of Arabidopsis infected by *Heterodera schachtii*. Plasmodesmata closure and transient cell isolation may serve to contain pathogen-derived effectors. Some of the effector proteins introduced by plant pathogens into host cells to counteract defence responses or to modify cellular metabolism to their own advantage can move through plasmodesmata, potentially to prepare host cells for infection [94,95].

Callose deposition/degradation around plasmodesmata necks or/and cell walls often occur during plant defence responses [96,97,98]. It is typically triggered by conserved PAMPs like fungal chitin and chitosan from the fungal cell wall [99]. Pectin-methyl-esterase and pectinase activities at plasmodesmata may contribute to local loosening of the wall to accommodate plasmodesmata aperture oscillations [95]. In this context, it seems likely that any invading microorganism—parasitic, mutualistic, or beneficial—would benefit from regulating plasmodesmata to maintain connectivity [100].

Host–pathogen interactions of the non-haustorial biotrophic fungi *Taphrina* spp. include an interface consisting of host and fungal cell walls, sometimes separated by a thin intercellular matrix, and are associated with an alteration of host plasma membrane permeability [75]. Electron microscopic images also demonstrate that the fan-shaped hyphae of *V. inaequalis* are embedded in a matrix-separating fungal cell wall and host plant cell wall, similar to an extrahaustorial matrix of biotrophic pathogens [101,102].

During the stroma development in the biotrophic grass endophyte *Epichloe amarillans*, the contacted host cell walls become modified to allow the easier acquisition of host nutrients by the fungus without the need for haustoria [103]. Changes in the permeability of the epidermal cell wall and plasma membrane are very likely, especially in areas of knob-like structures in fungal cells, transfer cells of plants, and fluorescent granules (exclusively underneath FH). The architecture of fan-shaped hyphae—small cells, thickness of anticlinal cell walls, high portion of vesicles, and membrane staples—corresponds to host transfer cells. The fluorescent granules underneath the core of FH are surrounded by a sealing lip from ultra-thin hyphae at the margin, which prevents the horizontal loss of nutrients. The increase in the density of fluorescent granules at the interface between epidermal cells and fan-shaped hyphae emphasises the dynamic nature of the interaction; however, it is not clear whether it is an indicator of increased or decreased transfer of nutrients and other compounds.

*V. inaequalis* secretes effectors in order to manipulate the metabolism/response of its host. Effectors may be directly released into plant cells/actively taken up by plant cells via endocytosis—symplastic localisation of a resistance protein coded by *Rvi15* has been reported by Schouten et al. [104]—or released into the apoplast, where they may travel longer distances and become active at or in plant cells remote from the infection site. Host cell re-programming by fungal effector molecules is likely to also cause morphological modifications of leaf cells.

Increased Release and Flow of Nutrients from Remote Plant Cells

The formation of globular protuberances of mesophyll cells in response to *V. inaequalis* infection has been already documented in the microscopic classification of scab symptoms [105] and in a textbook [106]; however, these authors had no explanation of their relevance and function or did not mention this phenomenon at all. Globules exuded by hypertrophied mesophyll cells towards intercellular spaces were observed in weakly susceptible host genotypes, whereas susceptible genotypes showed no morphological modifications of the mesophyll at the time of symptom appearance [105]. The globules could be stained with toluidine blue, which is used in plant specimens to detect pectin and lignin [107,108]. Ultrastructural information indicates a link between pit fields/plasmodesmata and the globules on the outside of mesophyll cells—the intimate contact between pathogens and epidermal cell walls hampers the detection of epidermal globules. As an activation of the plasmodesmal transport into the apoplast does not require the formation of cell wall protuberances into the intercellular space, this formation is likely to be associated with another order of magnitude of organic compound release from the symplast.

Pectinaceous beads are protuberances which occur on the outside of cell walls facing intercellular spaces. They are bead-like projections consisting of pectic polysaccharides, proteins, and fatty acids. These beads are a normal feature of epidermal and mesophyll cells of *Xyris* spp. leaves [109] on the surfaces of callus cells during grafting or wound healing, where pectin fragments (via pectinase activity) may function as signalling molecules for the compatibility/incompatibility of grafts, or on the outer surface of mesophyll cells in tobacco and some tree species [75]. The larger size and the lower number per cell distinguish the globules induced by *V. inaequalis* from those described in the literature so far, although a pectinaceous nature is in agreement with the release of pectinases by *V. inaequalis*.

Biotrophy and the Holomorph of *V. inaequalis*

The development of ontogenetic or adult plant resistance of fully expanded apple leaves to scab infection is in agreement with the transition from sink to source. As leaves age, plasmodesmal transport within the epidermal layer decreases gradually and quantitatively, and leaves switch from heterotrophic sinks for carbohydrates, nitrogen, and other resources to photoautotrophic sources of carbohydrates for the rest of the plant [110,111]. Restriction of plasmodesmata transport in source leaves strongly limits the fungal pathogen’s capacity to redirect the flow of nutrients. Under experimental conditions, latent infections with subcuticular growth but only rare sporulation may produce slightly chlorotic tissue and no penetration of the cuticle from the inside of the plant. Nevertheless, earlier, successful colonisation events are suitable to result in the formation of green islands on sab-infected leaves in late pathogenesis stages, demonstrating the pathogen effect on the phytohormone balance within leaf tissue typical for biotroph host–pathogen interactions.

Biotrophy depends on the capability of plant pathogens to prevent host defence reactions by modifications of surface properties and to manipulate the host cells to provide the organic nutrients required for growth and reproduction and is rather independent of structures like intracellular, still apoplastic haustoria. Other mechanisms like the redirection of water flow and the induction of plant transport mechanisms into the extracellular space in combination with a large extracellular interface may also be suitable. *V. inaequalis* uses the water vapour pressure deficit between plant tissue and ambient air as the driving force of nutrient flow from plant cells to the pathogen [2]. Nevertheless, this mechanism highly depends on the water status of the host and, thus, also on environmental conditions. Drought conditions result in precocious plant damage and the end of the biotroph stage, but *V. inaequalis* survives under these conditions and resumes activity on fallen leaves in autumn as a saprophyte to produce the sexual stage during hibernation. This type of biotroph lifestyle is well adapted to moderate climates with a sufficient water supply and medium temperatures limiting the overall rate of apple transpiration.

## 5. Conclusions

**Figure 10:**
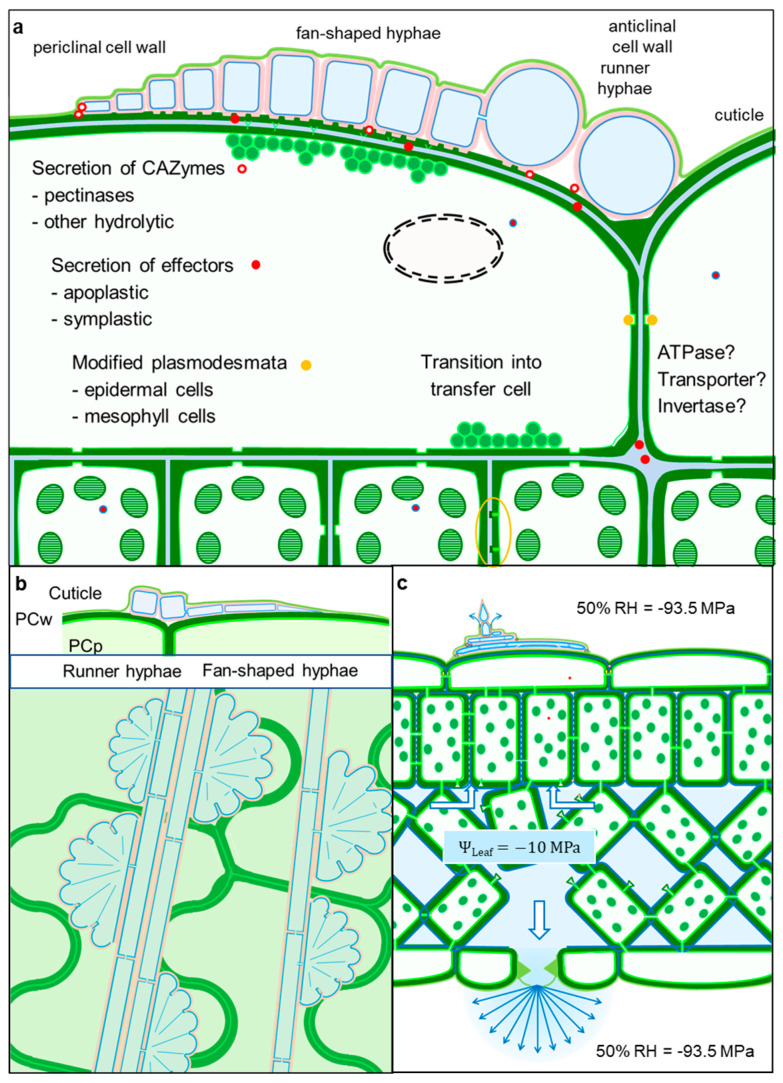
Model of *V. inaequalis* infection structures and apple leaf tissue responses. The primary stroma advances subcuticularly by the secretion of pectinolytic enzymes and forms runner hyphae for spreading; small and very thin fan-shaped hyphae cover large areas of the epidermal cell lumen for nutrient uptake. The secretion of effectors that may stay apoplastic or become symplastic contributes to modifications of epidermal and mesophyll cells. Intimate contact of fungal hyphae with the plant surface and partial degradation result in cell wall loosening and the formation of membranous cell wall appositions in responsive cells. Modifications of plasmodesmata in anticlinal pit fields favour the vertical flow of water and nutrients, which is strongly increased after the perforation of the cuticle due to conidiophore formation 5 to 6 d p.i. Secondary plasmodesmata at the plant–pathogen interface improve nutrient supply of the biotroph pathogen. Formation of globular protuberances on the surface of mesophyll cells (orange oval) increases the nutrition of the subcuticular fungus in the later stages of pathogenesis (**a**). Growth pattern of runner hyphae and lateral fan-shaped hyphae between cuticle and epidermal cells (**b**). Increased cuticular transpiration due to cuticle perforation by conidiophores redirects the flow of water and nutrient to the subcuticularly growing pathogen (**c**).

From our knowledge on the effect of scabs on the balance and movement of water in apple leaves [2] and the detailed studies on both subcuticular infection structures and morphological modifications of apple tissue in response to pathogen colonisation, a model of the lifestyle of *V. inaequalis* has been conceptualised (Figure 10). The pathogen takes up nutrients from living host cells by (I) intimate contact with a large interface between pathogen and host cells, provided by fan-shaped hyphae with small and very thin cells and facilitated by the presence of local vents in the fungal cell wall (Figure 10a,b); (II) induction of secondary plasmodesmata in the epidermal cell wall and blockage of anticlinal pit fields by callose accumulation, favouring the flow of nutrients (and effectors) in a vertical direction (Figure 10a); (III) fungal effectors modifying the permeability of host cells and causing the transition of some epidermal cells into transfer cells, as well as the formation of globular protuberances (from pit fields?) in mesophyll cells, which support an apoplastic nutrient flow in the direction of the subcuticular hyphae, driven by the water flow through the cuticular pores produced by conidiophores which penetrate the cuticle from the subcuticular space to the outside of the leaf (Figure 10a,c).

Non-obligate biotrophic pathogens may be cultivated on artificial media and live saprotrophically on dead leaf tissue in autumn. *V. inaequalis* produces conidia for polycyclic spread in its biotroph stage as long as young host tissue is available; the pathogen lacks a necrotroph stage with active killing of plant cells by enzymes or toxins but resumes growth with melanised hyphae saprotrophically only after dieback of leaf tissue due to senescence (Figure S3). Hemibiotrophy, therefore, has to be differentiated into the combinations biotrophy–necrotrophy and biotrophy–saprotrophy.

## Data Availability

The data supporting the findings of this study are available from the first author, Ulrike Steiner, upon reasonable request.

## Supplementary Materials

The following supporting information can be downloaded at https://www.mdpi.com/article/10.3390/jof10120831/s1, Figure S1: Subcuticular development of *Venturia inaequalis* hyphae; Figure S2: Reaction of apple tissue to subcuticular leaf colonisation by *Venturia inaequalis*; Figure S3: Life cycle and lifestyles of the holomorph of *Venturia inaequalis*.
