# Supplementary material for: The Hemibiotrophic Apple Scab Fungus Venturia inaequalis Induces a Biotrophic Interface but Lacks a Necrotrophic Stage
Source: J Fungi (Basel). 2024 Nov 29;10(12):831. doi: 10.3390/jof10120831 (PMC11676478; doi:10.3390/jof10120831)
Supplement: Supplementary file 1 [file jof-10-00831-s001.zip › jof-3285605-supplementary.pdf]

Supplementary Materials

Ulrike Steiner and Erich-Christian Oerke: The hemibiotrophic apple scab fungus *Venturia inaequalis* induces a biotrophic interface, but lacks a necrotrophic stage

Figure S1 Subcuticular development of *Venturia inaequalis* hyphae

Figure S2 Reaction of apple tissue to subcuticular leaf colonization by *Venturia inaequalis*.

Figure S3 Life cycle and life styles of the holomorph of *Venturia inaequalis*.

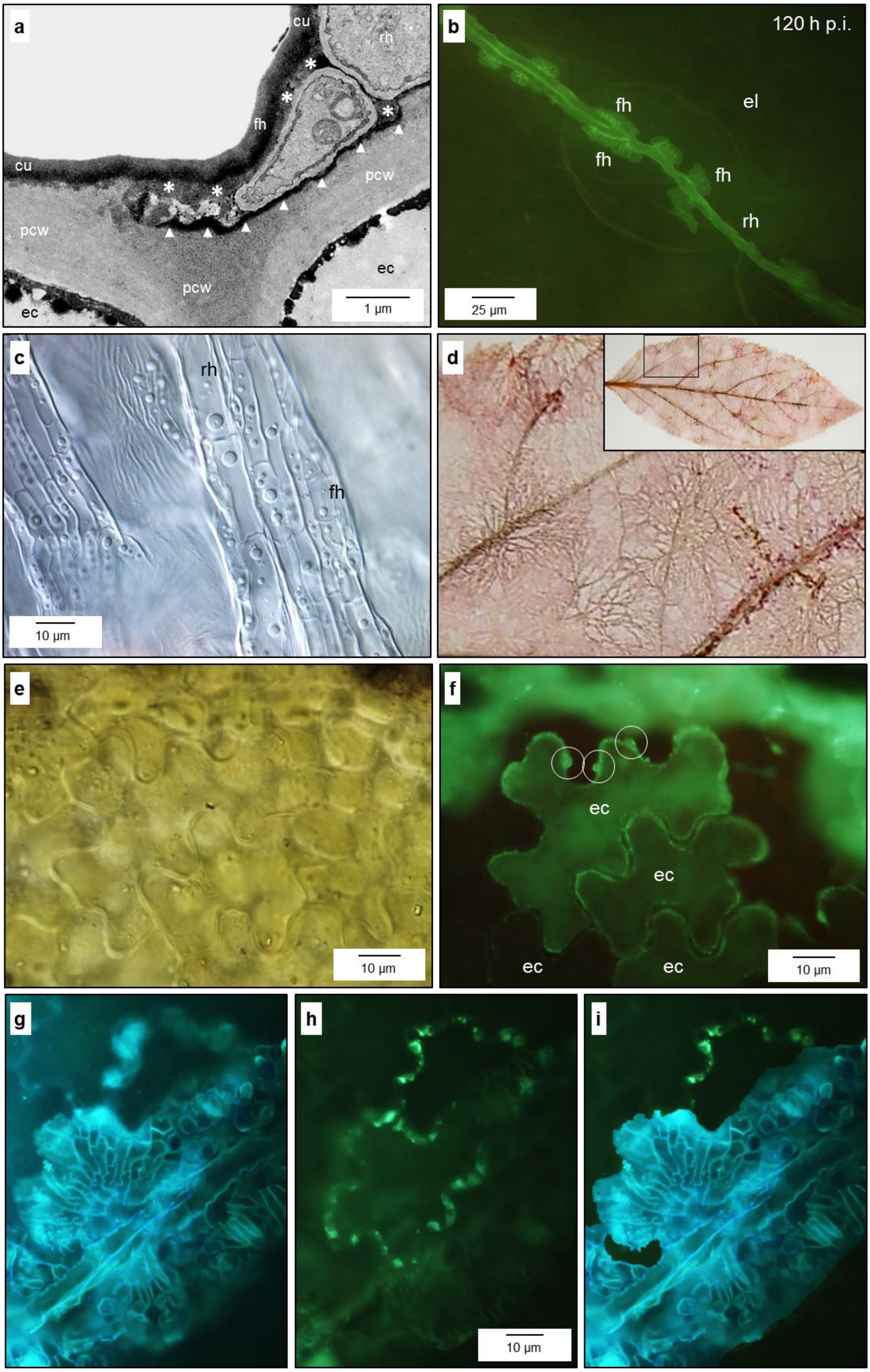

Figure S1 Subcuticular development of *Venturia inaequalis* hyphae. Runner hyphae (rh) and early stages of fan-shaped hyphae (fh) with knob-like structures in close contact with the epidermal layer (**a**); subcuticular growth of a hyphal tip with partial degradation of plant cell

wall (triangles) and deposition of material (asterisks) between cuticle (cu) and plant cell wall (pcw) of epidermal cells (ec; **b**); runner hyphae with early stages of fan-shaped hyphae above the epidermal layer (el; **c**); colonization of veins and intercostal leaf areas by subcuticular hyphae (**d**); formation of fluorescing granules (gr) at the interface between fan-shaped hyphae and epidermal cells (**e,f**; focus level of f a little bit lower than that of e); fan-shaped hyphae and multi-layered secondary stroma (ss; **g**); simple pore between two cells of a fungal hyphae, neighbouring hyphae are separated by two cell walls (**h**). Light microscopy (a, c, e-f; c, e-f, fluorescence after aniline staining), transmission electron microscopy (b, h), RGB image (d), scanning electron microscopy (g).

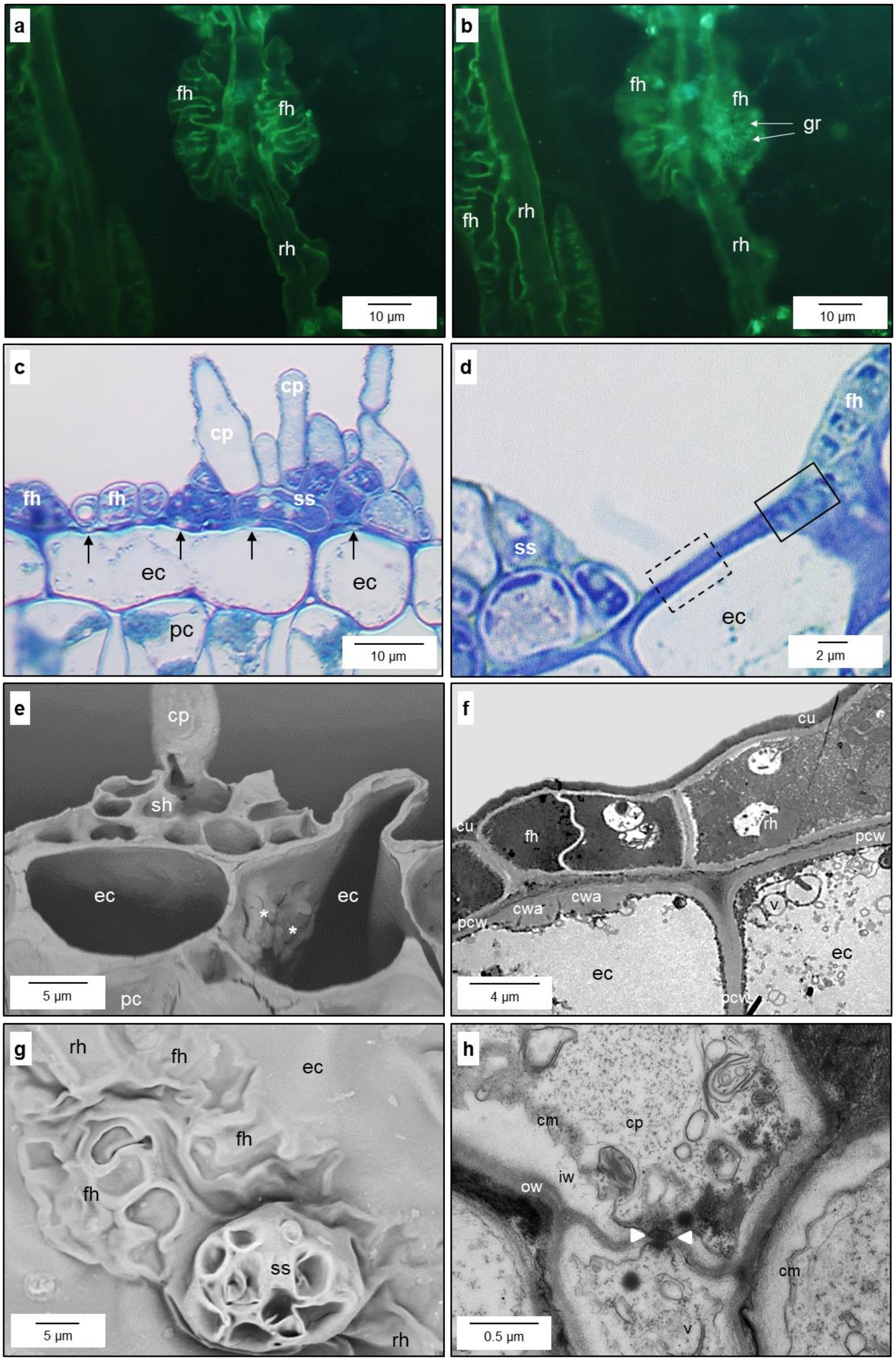

Figure S2 Reaction of apple tissue to subcuticular leaf colonization by *Venturia inaequalis*. Callose formation at anticlinal pit fields of epidermal cells (ec) in vicinity to subcuticular hyphae (sh; **a,b**); fluorescence of fan-shaped hyphae (**c**) and pit fields of epidermal cells (**d**) after aniline blue staining; overlay of c and d (**e**); loosening of epidermal cell wall (↑) beneath

secondary stroma (**f**); modifications of plant cell wall limited to the site of fungal colonization (**g**); formation of cell wall appositions in epidermal cells (**h,i**). Light microscopy (a-g; b-e, fluorescence; f-g, semi-thin sections stained with toluidine blue), scanning electron microscopy (h), transmission electron microscopy (i).

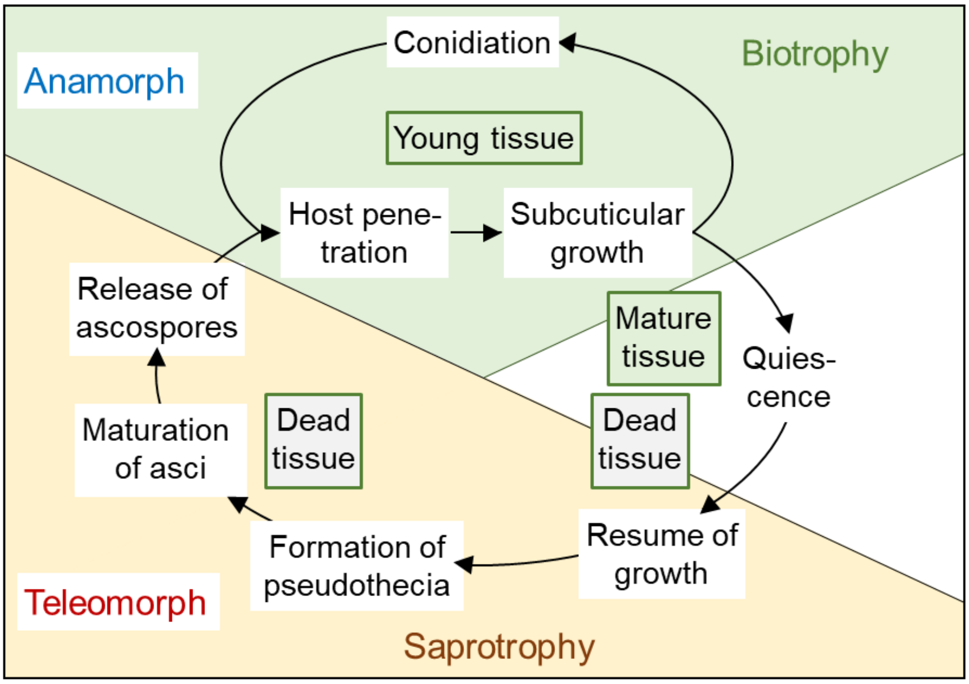

Figure S3 Life cycle and life styles of the holomorph of *Venturia inaequalis*.
